# A hybrid protein containing MSP1a repeats and Omp7, Omp8 and Omp9 epitopes protect immunized BALB/c mice against anaplasmosis

**DOI:** 10.1186/s13567-018-0503-4

**Published:** 2018-01-19

**Authors:** Alex Sander R. Cangussu, Luis André M. Mariúba, Pritesh Lalwani, Keila Dayane E. S. Pereira, Spartaco Astolphi-Filho, Patricia P. Orlandi, Sabrina Epiphanio, Kelvison F. Viana, Mucio Flavio B. Ribeiro, Hidelberto M. Silva, Claudio R. F. Marinho, Paulo A. Nogueira

**Affiliations:** 1 Universidade Federal do Amazonas–Programa de pós-graduação em Rede de Biodiversidade e Biotecnologia da Amazônia Legal, Manaus, AM/Brasil Brazil; 2 Universidade Federal do Tocantins–Engenharia de Bioprocessos e Biotecnologia, Gurupi, TO/Brazil Brazil; 3 Instituto Leônidas e Maria Deane-Fiocruz Amazônia, Manaus, Amazonas/Brazil Brazil; 4 Departamento de Análises Clínicas e Toxicológicas da Faculdade de Ciências Farmacêuticas, Universidade de São Paulo, São Paulo, São Paulo/Brazil Brazil; 5 Departamento de Parasitologia do Instituto de Ciências Biológicas, Universidade Federal de Minas Gerais, Belo Horizonte, Minas Gerais/Brazil Brazil; 6 Faculdade de Medicina, Centro Universitário Unirg, Gurupi, Tocantins/Brazil Brazil; 7 Departamento de Parasitologia do Instituto de Ciências Biomédicas, Universidade de São Paulo, São Paulo, Brazil

## Abstract

*Anaplasma marginale* (*A. marginale*) has a remarkable impact on livestock production, and an effective vaccine is not currently available due to the inexistence of a small animal model. Recently, BALB/c mice were successfully infected with *A. marginale,* resulting in an acute and persistent anaplasmosis infection. Here, we designed a hybrid protein containing repeats of polypeptide 1a from major surface protein-1 complex (MSP1a) repeats and common epitopes of outer membrane proteins (OMPs) OMP7, OMP8 and OMP9 expressed in *Escherichia coli*. Our proof-of-concept assessed vaccinal effectiveness against a challenge with live bacteria. The MSP1a/OMP7/8/9 immunized BALB/C mice exhibited a strong reduction in rickettsemia and had no signs of anaplasmosis or hepatic lesions. In contrast, the non-immunized mice exhibited signs of anaplasmosis and a body weight loss associated with increases in monocyte and neutrophil counts. Furthermore, the non-immunized mice displayed atrophies with chronic inflammatory infiltrates in the spleen and increased binucleation and hydropic degeneration in the hepatocytes. Our findings demonstrated that immunization with our hybrid protein induced a strong reduction in rickettsemia and conferred protection against anaplasmosis. Therefore, given the strong evidence of the protective effect against anaplasmosis, hybrid protein designs are potential candidates for the rational design of vaccinal subunits.

## Introduction

*Anaplasma marginale* (*A. marginale*) causes a life-threatening disease with a remarkable impact on human and animal health. Because tick vector control is difficult to achieve and an effective vaccine is nonexistent, anaplasmosis can cause severe economic losses in livestock production in tropical and subtropical regions [1–6]. *A. marginale* causes anaplasmosis in cattle of all ages, the severity increasing with age [7]. Following initial infection and 7–60 days incubation [1], the number of infected erythrocytes geometrically increases, doubling approximately every 24 h [8]. The invaded erythrocytes are phagocytozed by the reticuloendothelial system, causing progressive anemia [1, 7]. The clinical signs of anaplasmosis include fever, pale mucous membranes, anorexia, weight loss, decreased milk production, lethargy, icterus, gastrointestinal symptoms, miscarriage, and resultant death [1, 5, 9, 10]. Histopathological changes due to hypoxic conditions are observed in various organs. Marked thickening of the fat-containing liver capsule and bile retention causes hepatomegaly associated with hepatic hydropic degeneration. In the spleen, areas of lymphoid follicle atrophy associated with histiocytosis and fibrosis in the white pulp area are present [10].

A microscopic analysis is the best method for the diagnosis of anaplasmosis but is not reliable for detecting pre-symptomatic or carrier animals. In these cases, serological and molecular detection methods could be indicated [4]. The gold standard for the identification of *A. marginale*-free blood is the sub-inoculation of blood from the suspect animal into a splenectomized calf; however, this method is costly and raises animal welfare issues. Other methods, such as ELISA tests or PCR methods, have not been formally validated.

*Anaplasma marginale* establishes itself as a persistent infection that is difficult to control despite the routine use of preventive chemotherapy, but in cases of a carrier state in infected cattle, this intervention is a long-acting antimicrobial treatment [11]. Hence, new strategies are urgently needed for controlling disease spread [12]. Vaccination can be an ideal strategy for not only controlling disease transmission and spread but also for providing animals long-term immunity [2, 9–11, 13–16]. Currently, there are two commercially available vaccines for anaplasmosis; however, these vaccines are ineffective in controlling the epidemic and render animals as immune carriers, making disease control even more challenging. In addition, the presence of heterologous circulating strains and the risk associated with immunizing bulls or heifers with live vaccines enhance the necessity of the development of newer vaccines [17, 18]. Although *A. marginale* vaccines have been commercially available, chemotherapy remains the primary method of anaplasmosis control [11].

Additionally, cell surface proteins in *A. marginale* have been the focus of vaccine development since studies have shown that immunization with the purified outer membranes conferred protection against anaplasmosis [14, 19, 20]. Among the outer membrane proteins (OMPs), two major surface proteins belong to the MSP1 and MSP2 superfamilies [12, 21, 22]. The N-terminus portion of MSP1a contains tandem repeats that are conserved among *A. marginale* isolates, which have a sufficient effect on the adhesion to bovine erythrocytes and tick cells. The repeated sequences enhanced specific B and T cell responses and elicited neutralizing antibodies that conferred protection against anaplasmosis [14, 17, 23, 24]. Heterologous OMPs with conserved epitopes (OMP7–9) were identified using mass spectrometry with material adsorbed with serum IgG against surface complex vaccinates. The *omp7*–*9* genes belong to the msp2 superfamily and are arranged in tandem with a structure that is similar to that of a five-gene operon [14, 23]. OMP7 to OMP9 are invariant proteins that are known to be expressed at high levels in bovine erythrocytes, and they are components of the outer membrane protein complexes that are capable of inducing complete protection [14, 25].

Recently, BALB/c mice were successfully infected with *A. marginale*, similar to the pathogen *Anaplasma phagocytophilum* responsible for human granulocytic anaplasmosis [26–28]. In another study, following challenge by *Anaplasma* spp. isolated from natural hosts, the BALB/c mice showed injury and liver inflammation with a direct contribution by neutrophils [10, 29, 30].

Although the signs of anaplasmosis in the murine *A. marginale* model were not identical to those described in humans or ruminants, the BALB/c mice developed an acute and chronic infection [27, 28]. Furthermore, we were able to detect hepatocyte injury and liver inflammation with a direct contribution by neutrophils after a challenge with *Anaplasma s*pp. in natural hosts [10, 29, 30].

In the present study, we employed a murine *A. marginale* model to assess a hybrid protein containing epitopes of major surface protein 1a (MSP1a) and the outer membrane proteins (OMPs) 7–9 as a subunit vaccine against anaplasmosis. Based on clinical signs and histopathological criteria established in acute and chronic infection, our immunogen protected BALB/c mice challenged with *A. marginale* strain UFMG2.

## Materials and methods

### Mice

Seven-week-old female BALB/c mice were bred under pathogen-free conditions in our isogenic mouse facilities, in the Tocantins Federal University, (TO, Brazil). In all experiments, the welfare of the animals was taken in consideration. They were housed in a standard polycarbonate cage of 41 × 34 × 16 cm with wood shavings bedding, and a maximum of five mice per cage. We also attempted to reduce the stress of individual housing (when necessary) by environmental enrichment with nestlets and small play tunnels. The animal room was under controlled temperature and humidity in a light and dark cycle of 12 h. Mice had ad libitum access to food and water.

### Anesthesia and euthanasia

All efforts were made to prevent undue stress or pain to the mice. The mice were humanely euthanized once they show the following clinical signs: lethargy; hypothermia and/or difficulty of breathing. The mice were euthanized with ketamine (300 mg/kg) (Vetbrands, Brazil) and xylazine (22.5 mg/kg) (Syntec, Brazil). Consciousness was checked by testing the pedal reflex and observing heartbeats and breathing movements. All experiments were performed in accordance with the ethical guidelines for experiments with mice, and the protocols were approved by the National Council for Control of Animal Experimentation, Federal University of Tocantins Animal Experimentation Committee (CEUA No 23101.003595/2015-15). The guidelines for animal use and care were based on the standards established by The Brazilian College of Animal Experimentation (COBEA).

### *Anaplasma* UFMG2 infection and challenge

The strain *A. marginale* UFMG2 and tick cells were kindly provided by the Department of Parasitology, Institute of Biological Sciences, Federal University of Minas Gerais [30]. Briefly, the culture IDE8 tick cells were incubated in complete L15B culture medium (Sigma-Aldrich, Brazil), supplemented with 7% fetal bovine serum without antibiotics. The *A. marginale* UFMG2 was inoculated in the IDE8 cell culture, incubated at 34 °C for 15 days and monitored by microscopy using Giemsa staining. Then, the infected IDE8 cells were detached by trypsinization and washed to remove traces of trypsin. For the *A. marginale* release, the IDE8 cells were ruptured by shearing using a sterile insulin-like syringe. Three-week-old female BALB/c mice were divided into three groups. One group received three immunization series of the hybrid MSP1a/OMP7/9 protein mixed with the ISA adjuvant (immunized group; *N* = 5). The second group (*N* = 5) received only the ISA adjuvant (adjuvant group), and the third group (*N* = 5) received PBS (non-immunized group; *N* = 5). The mice were challenged with 3 × 10^5^ cells/mL of *A. marginale* UFMG2. All animals were monitored temperature and weight every day, fur alterations, as opacity and ruffle, and disturbed behavior, as aggressiveness or lethargy. The lethargy and weight loss were indicative of disease severity in the non-immunized mice. In the 42 day, adjuvant group presented high level of lethargy, then all animals were euthanized with an overdose of combining chemical anesthetics, as previously described [31].

### Selection of vaccine epitopes and protein construct

To design the hybrid, MSP1a/OMP7/8/9 were selected, and T- and B-cell epitopes were predicted from the MSP1a tandem repeats and the common epitopes of Omp7, Omp8 and Omp9 according to the antigenicity, flexibility and immune dominancy using the Immune epitope algorithm [32]. The 3D prediction was analyzing by the i-TASSER algorithm [33]. The in silico-designed synthetic hybrid MSP1a/Omp7/8/9 gene was flanked with 6-histidine nucleotide codons at the amino-terminus for purification by affinity chromatography. Furthermore, the hybrid MSP1a/Omp7/8/9 gene was codon optimized for expression in the *Escherichia coli* strain DH5αF’IQ and was obtained as a synthetic DNA double strand from IDT (Integrated DNA Technologies, USA). The synthetic DNA was then cloned into pGEM-T Easy (Promega) and sub-cloned into pHT43 (Mo_Bi_Tec). A chemical transformation was performed using CaCl_2_ in *E.* coli DH5αF’IQ, followed by incubation at 37 °C for 1 h, and raw MSP1a/OMP7/8/9 was obtained after culturing in isopropyl β-d-1-thiogalactopyranoside (IPTG) 1 mM, ampicillin, and an LB medium.

### Protein purification and vaccine formulation

A 6x-histidine tag was added to the synthetic MSP1a/omp7/8/9 protein at the amino-terminus, and the purification was performed using Nickel-labeled Sepharose affinity chromatography. Briefly, fifty milliliters of the bacterial growth of *E. coli* DH5αF’IQ transformed with pHT43-MSP1a/OMP7/9 were resuspended in MCAC buffer (20 mM Tris pH 7.9, 0.5% w/v NaCl and 10% v/v of glycerol). Twenty-five microliters of Triton-X100 were added to disrupt the membrane by freezing and thawing. After 10 000 × *g* spin for 20–30 min at 4 °C, the supernatant was filtered through a 0.22 µm membrane and purified with Ni–NTA superflow 6xHis tagged, according to protocol (Qiagen, Brazil). The purification was performed using an MCAC buffer adjusted to a pH of 8.0 for binding, pH of 6.0 for lavage and pH of 3.0 for elution. The MSP1a/OMP7/8/9 hybrid was emulsified in ISA adjuvant (MONTANIDE™ ISA 50 V2, Seppic, Brazil) with a final concentration of 0.144 μg/mL MSP1a/OMP7/8/9 in 22% aqueous and 78% oil ISA adjuvant. The aqueous-oil emulsion was prepared by shearing using sterile syringes connected by a silicone hose.

### Characterization and antigenicity of MSP1a/OMP7/8/9

The molecular weight and purity of MSP1a/OMP7/8/9 were characterized on SDS-PAGE gel, and the antigenicity was evaluated by western blotting with bovine anaplasmosis serum. After running on the 15% SDS-PAGE gel, the proteins were transferred to a nitrocellulose membrane following the Bio-Rad protocol (Bio-rad, Brasil). Serum from a calf infected with *A. marginale* was obtained from a farm located in the city of Gurupi at coordinates 11°43′45″S, 49°04′07″W in the State of Tocantins, Brazil. Serum from healthy calves was employed as a negative control and used with peroxidase-conjugated rabbit IgG- anti-Bovine (Sigma, Brazil) at 1/2000 and the 0.05% of the 3,3′-Diaminobenzidine substrate (DAB).

### Immunogenicity of the MSP1a/OMP7/8/9 recombinant

Seven-week-old female BALB/c mice were used to assess the immunogenicity of our protein. Briefly, the mice were sedated according to our standards for the use of sedation in animals. Mice in the immunized group received MSP1a/OMP7/8/9 emulsified in ISA adjuvant, the adjuvant group received ISA 50 V2, and the non-immunized group received PBS. A volume of 100 μL was administered intramuscularly at 0, 21, and 42 days. To evaluate and quantify the IgG anti-MSP1a/OMP7/8/9 antibody serum levels, 100 µL of blood was collected after sedation and analgesia by check puncture on days 0, 21, and 42. Sera from non-immunized mice were used to calculate a cut-off value for the ELISA.

### Enzyme linked immunosorbent assay (ELISA)

Animals received three immunizations with 20 day intervals between doses, and the serum IgG was obtained before each immunization. In brief, 4 μg/mL of hybrid MSP1a/OMP7/8/9 were sensitized overnight at 4 °C in 96-well microplates (Nunc MaxiSorp^®^). After blocking for 1 h with 2% casein-PBS buffer (blocking buffer) at room temperature, the plates were washed (4 times) with 0.05% Tween-20-PBS buffer. The serum was diluted at a ratio of 1:50 in the blocking buffer and incubated for 16 h at 4 °C. After washing, the anti-mouse IgG peroxidase conjugate (Sigma, USA) was used at a dilution of 1:10 000 in the blocking buffer. After washing, TMB (BD Biosciences, USA) was added for 15 min and blocked with 2.5 M H_2_SO_4_. For immunogenicity evaluation, the optical density (OD) was measured at 450 nm and the reactivity of sera from non-immunized mice were used to determine the cut-off for anti MSP1a/OMP7/8/9. First, the mean was calculated with OD values of sera collected of each animal of three groups before to start the immunization (*N* = 15). The variance among the OD values was estimated by standard deviation (SD). The cut-off was determined by equation cut-off = mean OD + 2 × SD. The values above the cutoff were considered positive.

### Hematological data, rickettsemia counts and quantification of spherocytes

Forty-two days after the challenge, blood samples were obtained for the leukogram analysis at the Pet Shop Dog Center Veterinary Clinic (Gurupi/TO—Brazil). The non-immunized mice were used as a reference for the leukogram data. The rickettsemia was counted by optical microscopy using Giemsa staining [12]. In addition to the rickettsemia, changes in the erythrocytes and impaired pigmentation (spherocytosis) were measured. The free *A. marginale* UFM2 was quantified by microscopy using Giemsa staining, and the challenge dose was established with 3 × 10^5^ cells/mL.

### Histopathology

The liver and spleen were removed by necropsy and were fixed in 10% buffered formalin for the histopathology analyses. The tissues were processed and embedded in paraffin, and each tissue was used for the H&E staining. The liver and spleen lesions were evaluated based on histopathology observed during chronic *A. marginale* infection. The liver alteration were characterized by binucleation and hydropic degeneration in hepatocytes accompanied by inflammation, hyperemia, perivasculitis, and necrosis. In the spleen, lymphoid follicle atrophy associated with histiocytosis and fibrosis in both white and red pulps indicated anaplasmosis. Signs of immunization were evidenced by periarteriolar and follicular hyperplasia in the white pulp. Thus, each animal of the experimental groups received a score according to the grade of the alteration of liver and spleen as follows: (0) absence; (1) discrete, up to 25% of the field of observation; (2) moderate, greater than 25%, but less than 50%, of the field of observation; and (3) strong, greater than 50% of the field of observation.

### Statistical analysis

The Kruskal–Wallis ANOVA test was used to compare the three groups (i.e., immunized, adjuvant and non-immunized mice). To test any overall differences between related means measure of body weight, mice were followed for 42 days post-infection, and compared by Repeated Measures ANOVA (RM-ANOVA). The survival percentage was calculated by Kaplan Meyer analysis. The following parameters were compared: the number of mice presenting symptoms, leukogram counts, and rickettsemia and spherocytes counts. For the histopathological data, we compared the means of the scores from the animals (*n* = 5) in each group as follows: non-immunized versus adjuvant and non-immunized versus immunized group. The IgG anti-MSP1a/OMP7/8/9 serum levels of the immunized, adjuvant and non-immunized groups were measured and compared using two-way ANOVA. A *p*-value < 0.05 with a 95% CI was considered significant.

## Results

### Structure, expression, antigenicity and immunogenicity of the hybrid MSP1a/OMP7/8/9

**Figure 1:**
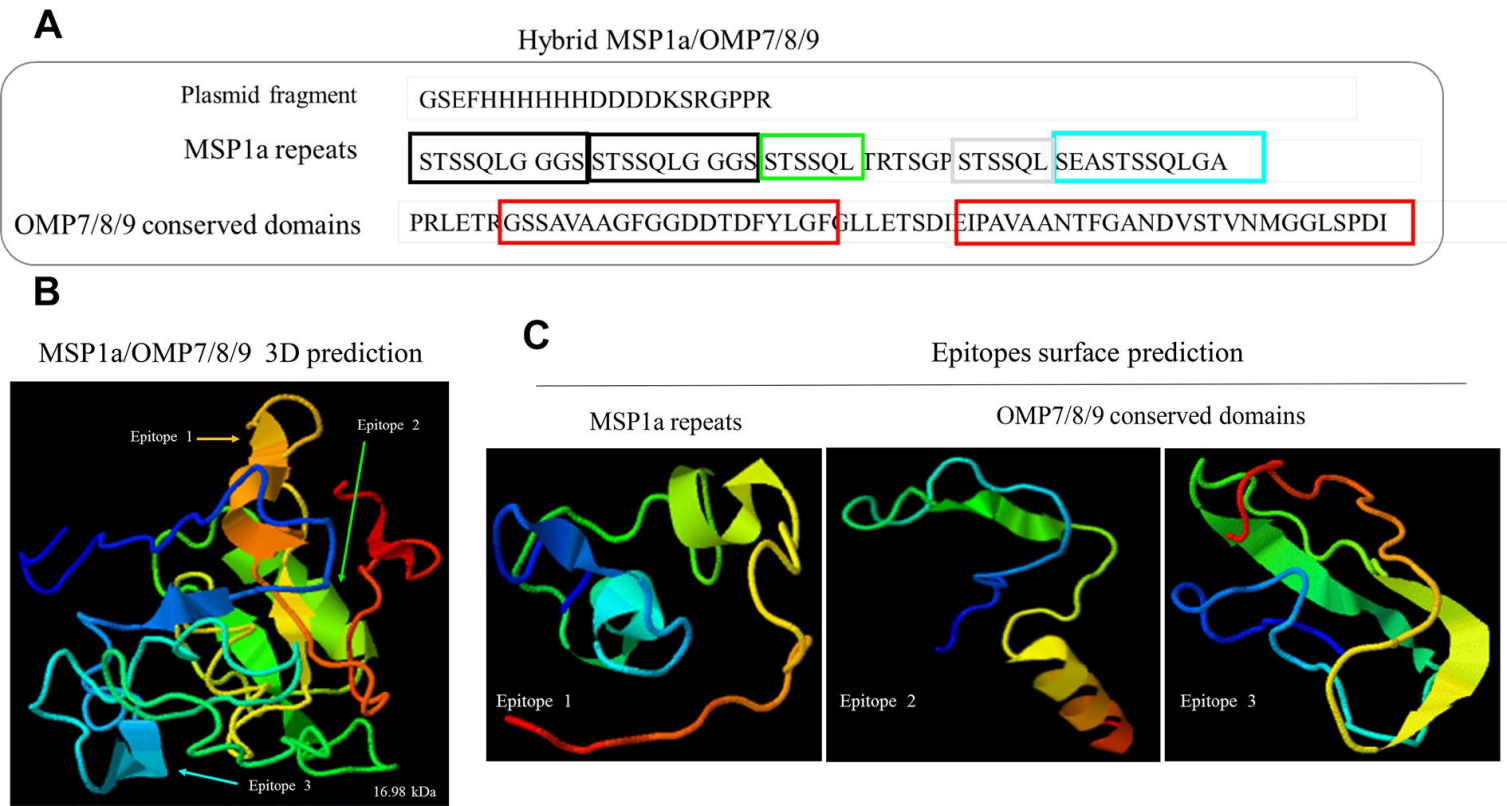
**Design of MSP1a/OMP7/8/9 protein. A** The amino acid sequence of the MSP1a/OMP7/8/9 protein containing MSP1a repeats and two common sequences from OMP7, OMP8 and OMP9. The synthetic DNA was designed using a sequence (GenBank: JN564640.1) from the *Anaplasma marginale* St. Maries strain. **B** The 3D structure of the MSP1a/OMP7/8/9 protein was predicted using the i-TASSER algorithm [33]. According to the 3D prediction, the MSP1a repeats and two common sequences were exposed on the surface of the recombinant protein. **C** Each panel shows in detail the secondary structure of the MSP1a repeats (left panel) and two common sequences (middle and right panels).

The MSP1a/OMP7/8/9 synthetic DNA sequence was designed with repeats in MSP1a and two common sequences from OMP7, OMP8 and OMP9 using *A. marginale* GenBank: JN564640.1 St. Maries. From MSP1a, we inserted STSSQLGGS (*n* = 2), STSSQL (*n* = 1) and one known sequence SEASTSSQLGA (*n* = 1) located between amino acids 15° to 153° of *msp1* gene. We used two common sequences, GSSAVAAGFGGDDTDFYLGFG and EIPAVAANTFGANDVSTVNMGGLSPDI, from OMP7, OMP8 and OMP9 located at position 1–58 of *omp7* gene (Figure 1A). The synthetic MSP1a/OMP7/8/9 sequence was inserted into a 6-his-tag plasmid for purification. The 3D prediction suggested that the MSP1 repeats and common OMP7–OMP9 surface antigens were exposed on the surface of the hybrid protein (Figure 1B). In detail, the cloned motifs displayed tertiary conformations (Figure 1C).

**Figure 2:**
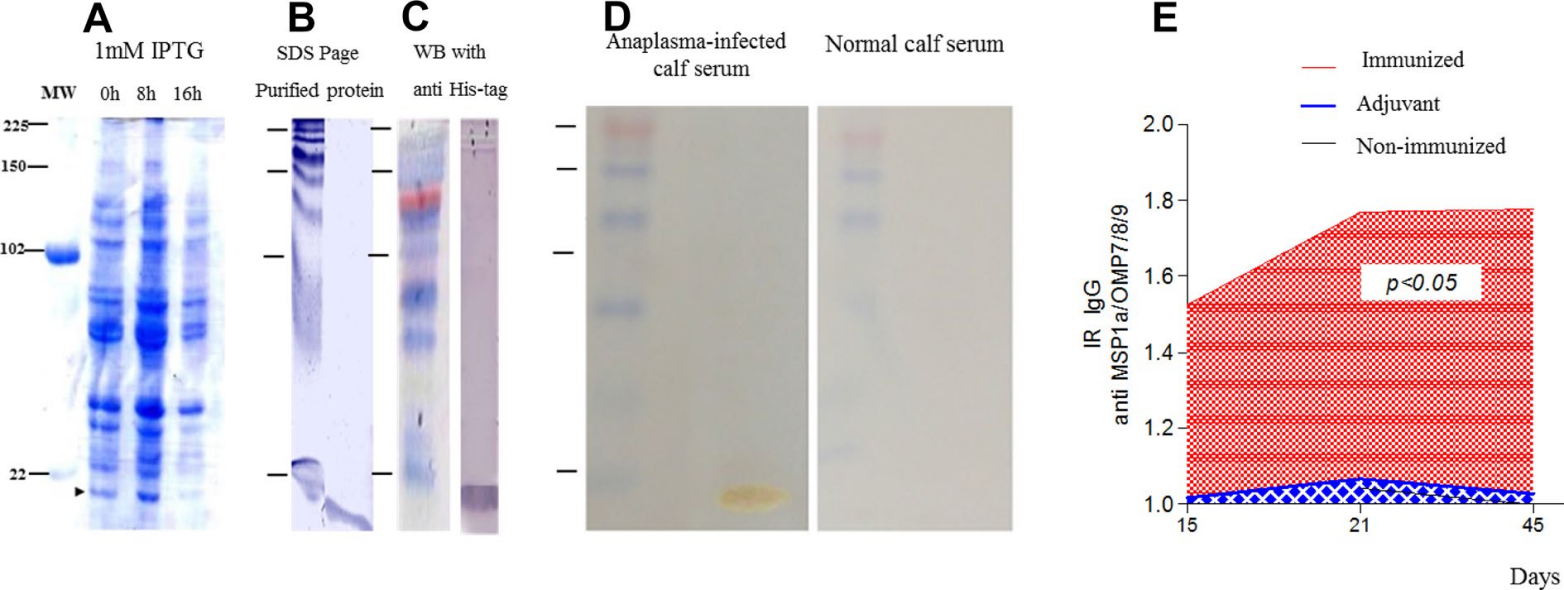
**Immunogenicity of the hybrid protein MSP1a/OMP7/9. A** Expression of the hybrid MSP1a/OMP7/9 protein after IPTG induction. Left panel: Supernatants of the *E. coli* strain DH5αF’IQ growth on 15% SDS-polyacrylamide gel electrophoresis (SDS-PAGE). Line 1: without IPTG at 0 h; line 2: after 8 h with IPTG; and line 3: 16 h after IPTG. MW—Molecular weight. **B** SDS-PAGE of hybrid MSP1a/OMP7/9 protein by affinity chromatography using Ni–NTA superflow 6xHis tagged (Qiagen, Brazil) (For detail, see “Materials and methods”). The purification was performed using an MCAC buffer (20 mM tris pH 7.9, 0.5% w/v NaCl and 10% v/v of glycerol), adjusted in pH 8.0 for binding, pH 6.0 for lavage and pH 3.0 for elution. **C** Western blotting (WB) with anti-HisG (Invitrogen) to characterize the molecular weight of the hybrid protein. **D** Western blotting (WB) of the hybrid protein to determine the antigenicity from anaplasmosis infected calf serum (left panel) and normal calf serum (right panel). **E** Immunogenicity of hybrid protein MSP1a/OMP7/9 after three immunization series emulsified with ISA adjuvant (MONTANIDE™ ISA 50 V2, Seppic, Brazil).

The in silico analysis suggested that the MSP1a/OMP7/8/9 protein was 17 kDa (ProtParam software), which was confirmed after its expression (Figure 2A) and purification on SDS/PAGE (Figure 2B). In addition, we confirmed that the purified protein was expressed as a hisTag by immunoblotting with an anti-hisTag antibody (Figure 2C). Then, the immunoblotting and probing of serum from animals with anaplasmosis confirmed the MSP1a/OMP7/8/9 antigenicity in comparison to the healthy calf serum, which served as a negative control (Figure 2D). Furthermore, after immunizing the mice with three doses of the protein, the immunogenicity was confirmed by measuring the anti-MSP1a/OMP7/8/9 IgG antibodies by ELISA. The high antibody titers confirm the immunogenicity and suitability of this antigen as a vaccine candidate (Figure 2E).

### In vivo challenge with *A. marginale* in immunized mice

**Figure 3:**
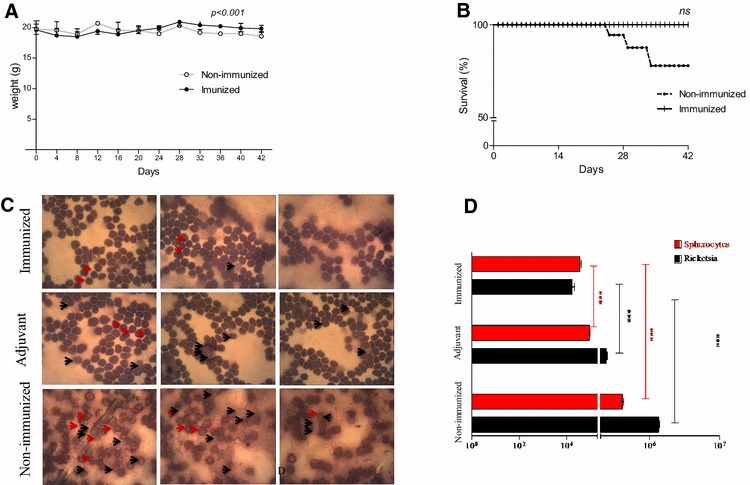
**Challenge with the** ***A. marginale*** **strain UFMG2 in mice**. Three-week-old female BALB/c mice were divided into three groups. One group received three immunization series of the hybrid MSP1a/OMP7/9 protein mixed with the ISA adjuvant (immunized group; *N* = 5). The second group (*N* = 5) received only the ISA adjuvant (Adjuvant group), and the third group (*N* = 5) received PBS (non-immunized group; *N* = 5). The mice were challenged with 3 × 10^5^ cells/mL of *A. marginale* UFMG2. The animals were observed for 42 days to measure weight loss, deaths, and signs, such as lethargy and ruffled and opaque fur (see Table 1). **A** Differences between related means of body weight followed for 42 days post-infection, the RM-ANOVA Test showed differences between the immunized and non-immunized mice showed differences between the immunized and non-immunized mice. The difference was evaluated by two-way RM ANOVA. The adjuvant group did not differ from either group (not shown). **B** The immunized group had 100% survival while 60% of non-immunized mice survived in Kaplan Meyer analysis. The adjuvant group did not differ from the immunized mice (not showing). **C** Morphological changes, such as cell destruction and pigmentation loss and featuring bacterial invasion in infected erythrocytes at 48 days post-infection. **D** Rickettsemia and *A. marginale* load and spherocytes counts were determined with blood smears stained with hematoxylin–eosin by optical microscopy [10].

Three groups of mice were challenged with 3 × 10^5^ cells/mL of *A. marginale* UFMG2a and were observed for 42 days for anaplasmosis symptoms, such as weight loss, lethargy, ruffled and/or opaque fur and death (Table 1). Lethargy and weight loss evidenced severity of disease in the non-immunized group. In the 42 day, adjuvant group presented lethargy, hence the survivors’ animals were euthanized. The immunized group did not display any symptoms, while the subclinical dose was able to cause body weight loss in the non-immunized group after 30 day (*p* < 0.001), which coincided with the naturally occurrence of acute anaplasmosis (Figure 3A). Additionally, the overall coefficient of variation of the body weights of the immunized mice was low compared to that of the non-immunized group. The body weight loss in the adjuvant group was intermediary to both groups without a significant difference (data not shown). Nonetheless, ruffled and opaque fur was evident in the surviving mice in the non-immunized and adjuvant group (Table 1). The mice immunized with the hybrid MSP1a/OMP7/8/9 protein showed better weight control and no clinical presentation of anaplasmosis compared with the non-immunized mice (Figure 3B).

**Table 1:** **Comparison between signals and leukogram data between MSP1a/OMP7/8/9 protein-immunized mice and controls mice**

	Immunized	Adjuvant	Non-immunized	*p*	Reference
Symptoms, animals with symptom (Total of animals)^a^					
Opaque fur	0 (5)	5 (5)	5 (5)	nd	0 (5)
Ruffled fur	0 (5)	5 (5)	5 (5)	nd	0 (5)
Weight loss	0 (5)	0 (5)	5 (5)	nd	0 (5)
Lethargy	0 (5)	5 (5)	5 (5)	nd	0 (5)
Death	0 (5)	0 (5)	2 (5)	nd	0 (5)
Leukogram^b^					
Leucocytes	5600.0 (529.2)*	7266.7 (1814.8)	7733.3 (1222.0)*	*0.0415*	3483.3 (2119.7)
Lymphocytes	2053.5 (835.3)	2640.0 (254.4)	2242.7 (671.0)	0.3967	2223.4 (778.2)
Monocytes	130.0 (29.5)*	180.7 (112.9)	253.3 (12.2)*	*0.0071*	210.7 (27.2)
Neutrophils	3318.7 (370.1)*	4457.3 (1411.7)	5104.0 (595.9)*	*0.0380*	2014.0 (1396.4)
Red cells, millions/mm^3^	5.6 (0.5)	5.5 (0.9)	6.4 (0.6)	0.0631	5.6 (0.6)
Hemoglobin g/dL	16.8 (1.4)	16.7 (2.8)	19.5 (1.9)	0.1324	16.9 (1.8)
Hematocrit %	50.3 (4.0)	50.3 (8.3)	58.7 (5.8)	0.1303	50.7 (5.5)
MCV/mm^3^	90.3 (0.6)	90.7 (0.6)	91.0 (0.0)	0.2605	90.3 (0.6)
MHV/mm^3^	30.0 (0.0)	30.0 (0.0)	30.0 (0.0)	0.9999	30.0 (0.0)
MCHC/mm^3^	33.0 (0.0)	33.0 (0.0)	33.0 (0.0)	0.9999	33.0 (0.0)
Platelets, millions/mm^3^	323.7 (97.0)	403.3 (139.5)	281.3 (138.6)	0.6447	309.7 (45.6)
Creatinine, mg/dL	1.2 (0.5)	1.2 (0.4)	1.2 (0.5)	0.9854	1.1 (0.5)
Urea, mg/dL	56.3 (33.6)	55.0 (8.9)	52.0 (18.5)	0.9999	61.3 (20.4)

nd: not done.

* significance found by Kruskal Wallis T-test.

^a^Animals with symptom (Total of animals).

^b^For leukogram data, only three animals have been used in each parameter. Mean (standard deviation).

At 42 day after the challenge, the rickettsemia and spherocytes were quantified using optical microscopy to detect cell free rickettsia and infected erythrocyte cells (Figures 3C and D). The high intracellular invasion and cell destruction indicated rickettsia in the non-immunized group (Figure 3C). In addition, we observed a 100-fold increase in the rickettsemia in the non-immunized group compared to the immunized group (Figure 3D).

The rickettsemia was significantly lower in the immunized group than that in the adjuvant group (*p* < 0.001), while that the rickettsemia observed in the adjuvant group also was different compared to the non-immunized mice (Figure 3D). The morphological changes in the infected erythrocytes were compared, and the immunized group had a fewer number of spherocytes that the adjuvant and non-immunized group (*p* < 0.0001). In summary, the immunized mice exhibited a control of the *A. marginale* infection at subclinical levels and were protected against anaplasmosis.

### Hematological and histopathological presentation in the control and immunized mice

To assess with the effects of an *A. marginale* infection in mice, we performed complete blood counts and assessed the morphological alterations in the spleen and liver 42 day after the challenge. The immunized mice had moderate leukocytosis (5600 ± 529; *p* < 0.05). No significant difference in the number of lymphocytes was observed among the three experimental groups. However, increases in the neutrophils were observed in the non-immunized and adjuvant group compared to immunized group. The immunized group had lower monocyte counts after the *A. marginale* infection while weight loss, lethargy, ruffled and/or opaque fur and death were observed in the non-immunized and adjuvant group (Table 1). Overall, neutrophils and monocytes participated in the disease pathology and were important in protecting the mice against anaplasmosis.

**Figure 4:**
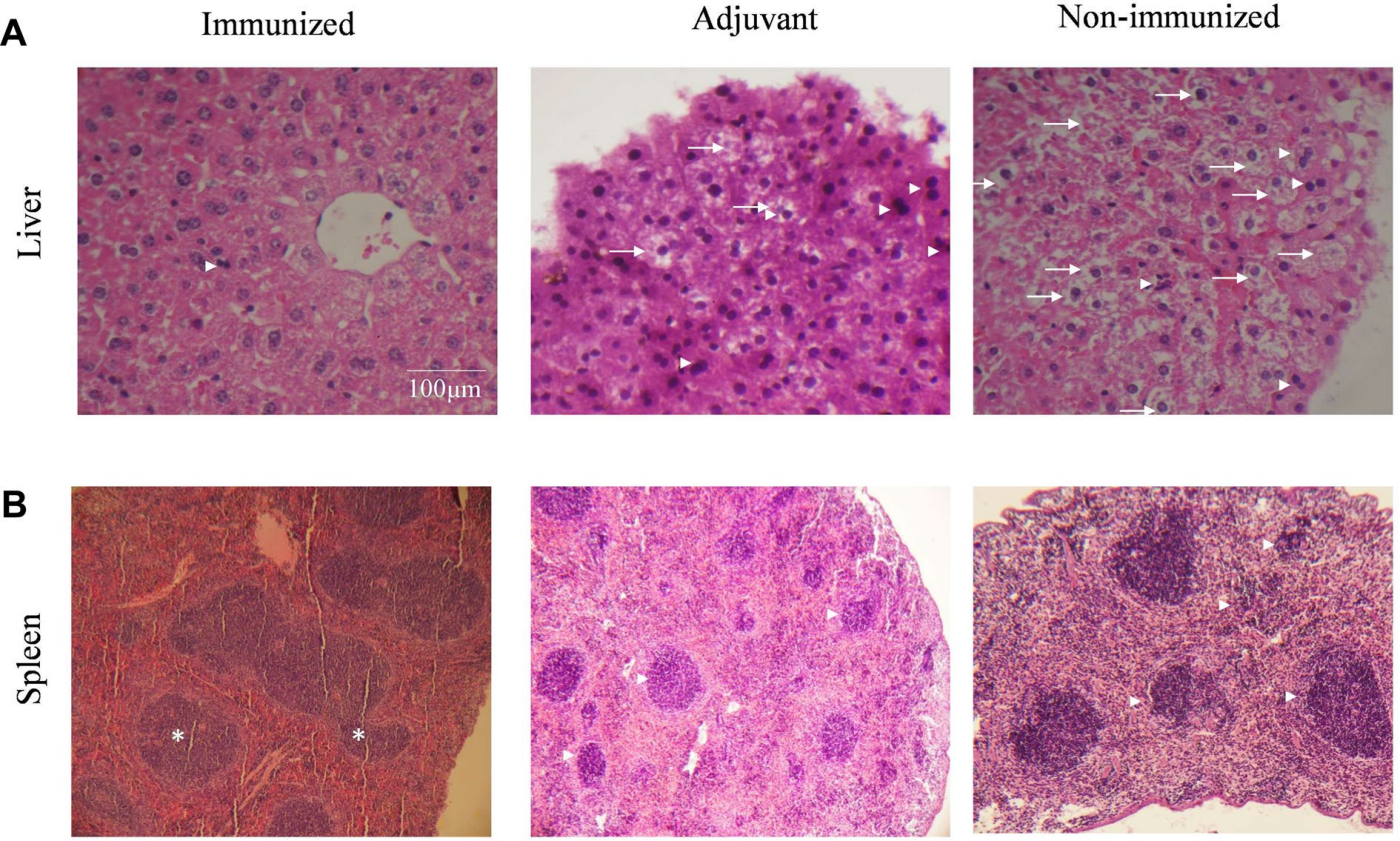
**Liver and spleen modifications after the** ***A. marginale*** **challenge.** All groups were challenged with 3 × 10^5^ cells/mL *A. marginale* UFMG2. **A** The liver histopathology shows hepatocytes binucleation (arrow head) and hydropic degeneration (arrow). **B** The spleen analysis indicates follicular hyperplasia in the white pulp (asterisk). In contrast, the adjuvant and non-immunized group showed lymphoid follicle atrophies (arrow head). Hematoxylin and eosin staining and scale bar = 100 μm.

To assess the mechanism by which the hybrid MSP1a/OMP7/8/9 protein provided protection in the immunized mice, we performed a pathological analysis of the liver and spleen. A summary of the histopathological analysis of the liver and spleen scored by comparing to the uninfected mice is provided in Table 2. The infection caused binucleated hepatocytes in the three groups, however non-immunized group displayed intense areas associated with perivasculitis, hyperemia and necrosis (Figure 4A). The binucleated hepatocytes areas in adjuvant group were more numerous than in immunized group. Nonetheless, the scores did not differ statistically. Another histopathological manifestation, the hydropic degeneration occurred only in the non-immunized group. The hydropic degeneration was well defined as cells without cytoplasm organization by central zones of the hepatic lobules. In the immunized mice, the hydropic degeneration score was minimal, while was intermediate in the adjuvant group. In the spleen, only the immunized group showed significant stimuli in the white pulp, and periarteriolar and follicular hyperplasia was well defined (Table 2). The white pulp and follicular area expansions demonstrate a consequence of the antigenic stimulation and subsequent proliferation of B cells (Figure 4B). These modifications were not present in the control and adjuvant group, implicating lymphoid follicle atrophies.

**Table 2:** **Histological changes in the spleen of MSP1a/OMP7/8/9 protein-immunized mice and controls mice**

	LIVER		SPLEEN					
	Hydrotropic degeneration		White pulp hyperplasia		Periarteriolar hyperplasia		Follicular hyperplasia	
	95% CI	*p*	95% CI	*p*	95% CI	*p*	95% CI	*p*
Non-immunized vs. immunized	0.2695 to 1.731	**	−2.397 to −0.9361	**	−2.064 to −0.6028	**	−2.397 to −0.9361	**
Non-immunized vs. adjuvant	−0.7305 to 0.7305	ns	−0.7305 to 0.7305	ns	−0.7305 to 0.7305	ns	−1.397 to 0.06387	ns

ns: not significant.

** *p* < 0.005.

## Discussion

Although vaccination is the best strategy against anaplasmosis, the high production cost, rickettsial genetic diversity, and risk associated with live vaccines impose great difficulties on anaplasmosis control [1, 25, 35, 36]. Thus, new strategies are needed for rationale vaccine development. The genus *Anaplasma,* belonging to order Rickettsiales, comprise species that are causative of tick-borne diseases and have a remarkable impact on human and animal health [1, 35, 37]. Previously, the major pitfall in anaplasmosis research was the lack of a suitable small animal model. However, several recent studies have demonstrated that the histopathological changes associated with acute and chronic *A. marginale* infections can be reproduced in murine models [26, 27, 38, 39].

Here, we have also successfully infected BALB/c mice with *A. marginale* UFMG2 released from IDE8 after mechanic rupture. Lethargy and weight loss evidenced severity of disease in mice non immunized and in mice of adjuvant groups with lethargy at 42 days after infection. We also evaluated hematological status and manifestations of disease in liver and spleen by histopathology examination.

Despite the use of a subclinical dose, the non-immunized mice developed liver lesions and displayed increases in the WBC, neutrophil and monocyte counts. With persistent infection, these mice displayed spherocytes as a consequence of infected erythrocytes and hepatic lesions, which is similar to the murine model of human granulocytic anaplasmosis [27, 29]. It has been established that neutrophils could be important mediators of innate responses, but under aberrant activation, they cause inflammatory hepatic lesions [10, 40, 41]. Consistently, the non-immunized group displayed increases in the neutrophil counts during the chronic infection that were concomitant with an intense hydropic degeneration throughout the hepatic portal and central zones [29]. Moreover, these mice displayed intense areas with binucleated hepatocytes associated with perivasculitis, hyperemia and necrosis, as observed in classical anaplasmosis models [10, 42]. Adjuvant group displayed slight histopathological manifestations. Hydropic degeneration occurred exclusively in these mice, which is consistent with several of the hepatic lesions observed in murine models of human monocytic ehrlichiosis [41]. Despite a subclinical dose with the highly fatal *A. marginale* strain, no liver lesions or changes were observed in the WBC count of immunized mice. These findings indicated conferred protection against anaplasmosis after immunization with hybrid protein. In contrast, the spleen from the non-immunized group displayed a discrete splenomegaly with few inflammatory infiltrates and focused follicular atrophy in the white pulp, which is a characteristic of spleen histopathology that is common in calves [10, 29, 43]. However, our immunized group displayed hyperplasia in the white pulp characterized by increased cellularity of larger, rounded and regular nuclear membranes and even paler stained cells, which is suggestive of a response to antigenic stimuli [43, 44].

Evidence has shown that the MSP-1 complex and both MSP-la and MSP-lb individually mediate adherence to bovine erythrocytes, and the antibody response against these proteins could block parasite invasion of erythrocytes and disease spread [1, 37, 45–47]. However, antigenic variability has hampered *A. marginale* vaccine development, whereas immunization with single antigens has shown partial or little protection against an *A. marginale* challenge [1, 12, 48, 49]. In this study, we have overcome this problem by creating a hybrid protein with immunodominant epitopes of several important surface protein; in addition, we also considered the antigenic variability for an efficient immune response [12, 18, 26, 46, 47, 50, 51]. Our hybrid protein contained motifs of a single MSP1a from the most characterized protein of the *A. marginale* MSPs [15] and three little-known OMP7–9 located in a putative operon from the *msp2* superfamily [22]. We designed, in tandem, longer sequences of the MSP1a repeats (STSSQLGGS and SEASTSSQLGA) with two common sequences of OMP7, OMP8 and OMP9 (GSSAVAAGFGGDDTDFYLGFG and EIPAVAANTFGANDVSTVNMGGLSPDI), and identified invariant and potential vaccine candidates that are highly expressed in bovine *A. marginale*-infected erythrocytes [14, 18, 20, 22, 25, 26, 35, 52]. The 3D prediction analysis suggested that our design allowed for the exposition of these motifs on the recombinant protein surface.

In the present study, our proof-of-concept was that a hybrid protein containing several epitopes of different antigens could protect mice against a challenge with a live and highly virulent *A. marginale* strain (UFMG2) [26, 34]. The immunized BALB/c mice did not exhibit signs of anaplasmosis, and the survival rate was 100%, while the non-immunized and adjuvant groups presented ruffled and opaque fur and considerable weight losses after the challenge. The serological responses after the immunization showed a considerably higher immunogenicity for MSP1a/OMP7/8/9, indicating that the protection against anaplasmosis may be associated with the elicitation of effector functions of humoral and cellular immune responses [18, 26]. Microscopically, we also demonstrated, for the first time, that the immunized group did not present damage in the red blood cells nor hepatic lesions. Thus, the prominent germinal centers in the secondary follicles in the immunized mice indicated that our hybrid protein elicited an effector humoral response that allowed us to evaluate the protection against *A. marginale* [18, 26, 47, 53].

In summary, we demonstrated a useful murine model to study *A. marginale* infection and design a recombinant protein containing potential epitopes of different antigens. Our hybrid protein immunization induced a strong reduction in rickettsemia and conferred protection against anaplasmosis. Thus, we provided strong protection against anaplasmosis with a hybrid protein, and therefore, other hybrid proteins should cover the antigenic diversity of *A. marginale* to achieve candidate subunit for anaplasmosis vaccine.

## Abbreviations

COBEA: Brazilian College of Animal Experimentation
*A. marginale* UFM2: virulent strain of cattle isolated by Prof. Dr. Mucio Ribeiro of the Federal University of Minas Gerais (UFMG strain number 2)
IDE8: cell lines cell line *Ixodes scapularis*
RM ANOVA: repeated measures ANOVA
ISA: adjuvants for Water-in-Oil (W/O) MONTANIDE SEPPIC ISA 50 V2
MSP2: major surface protein 2
MSP1a: major surface protein 1a
OMP7/8/9: outer membrane protein 7, 8 and 9
